# Genome sequence of cluster EK1 *Microbacterium foliorum* phage TownLake

**DOI:** 10.1128/mra.00405-26

**Published:** 2026-07-22

**Authors:** Angela Dawson, Aaron Jackson, Carlos Perez, Catalina Mercado, Isabela Duran Osorio, Samantha N. Cerit, Sandra M. Saji, Yamil Moya-Manganelly, Lara Becerra-Reymundo, Jaime Mayoral, Patricia Waikel

**Affiliations:** 1 Department of Biological Sciences, Florida International University 5450, Miami, Florida, USA; Queens College Department of Biology, Queens, New York, USA

**Keywords:** bacteriophages

## Abstract

Bacteriophage TownLake was discovered and isolated from an environmental sample in Pembroke Pines, Florida, using *Microbacterium foliorum* NRRL B-24224 as the host. TownLake has a 53,817 bp genome containing 52 predicted protein-coding genes and is assigned to subcluster EK1. Transmission electron microscopy revealed a podoviral morphology.

## ANNOUNCEMENT

Bacteriophages are a diverse group of viruses found in terrestrial, aquatic, and host-associated ecosystems. They shape microbial communities and contribute to nutrient cycling. Discovery and characterization are essential for understanding phage diversity, evolution, and host–phage interactions (1).

**Fig 1:**
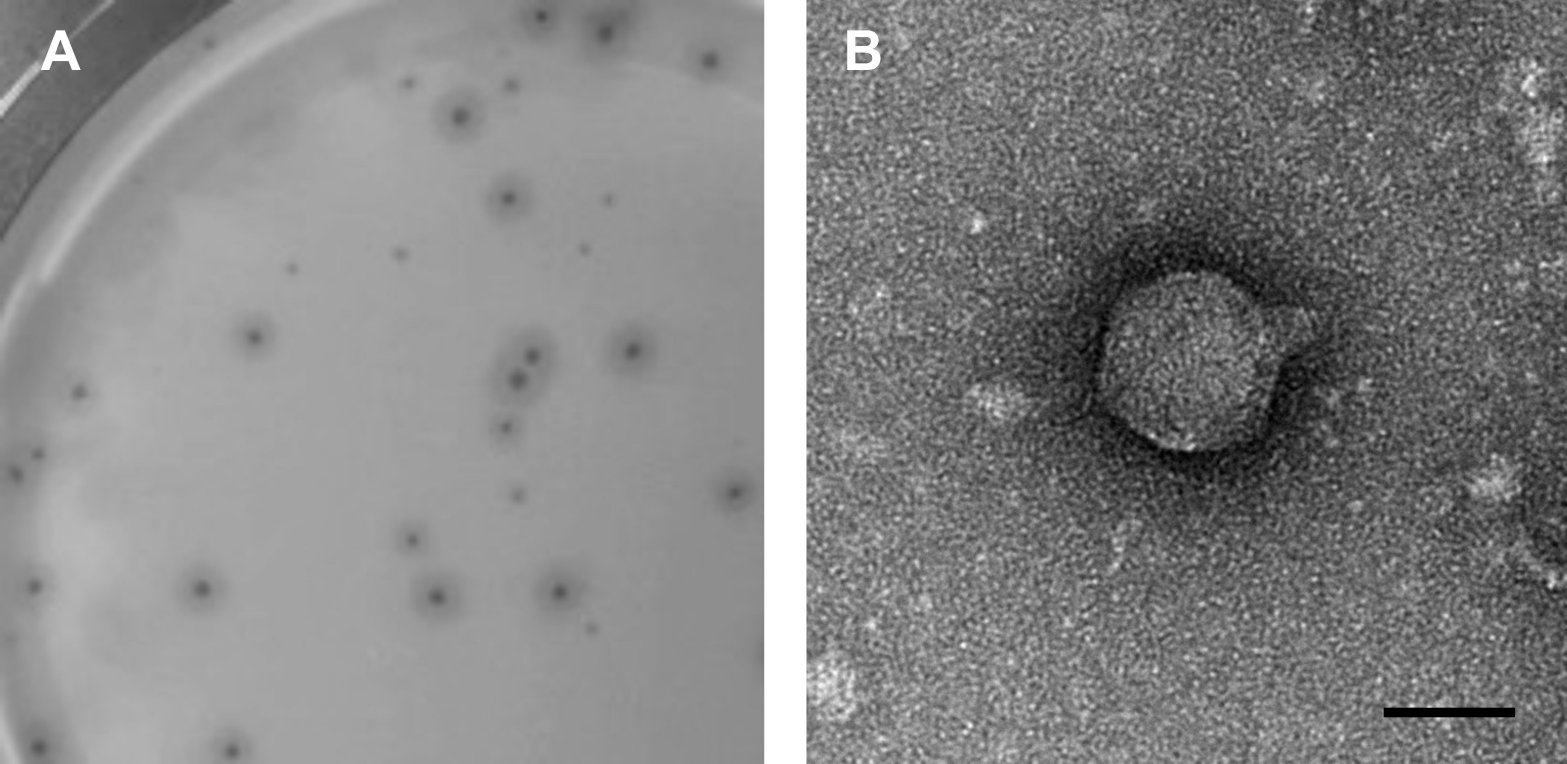
(**A**) Plaque assay of bacteriophage TownLake showing clear plaques with halo edges 4.28 ± 1.36 mm (*N* = 10) formed on *Microbacterium foliorum* NRRL B-24224 following incubation of 24 h at 30°C. Plate is 80 mm in diameter. (**B**) Transmission electron micrograph of bacteriophage TownLake demonstrating podoviral morphology, including an icosahedral capsid 65.97 ± 0.47 nm in diameter (*N* = 3). Scale bar, 50 nm. Phage samples were placed on 200-mesh formvar-covered, carbon-coated copper grids (EMS, Hatfield, PA, USA), incubated for 1 min, rinsed with ultrapure water, and stained with 2% uranyl acetate. Images were taken at 100 kV using a Hitachi HT7800 120 kV TEM with an AMT NanoSprint15B digital camera.

TownLake was isolated from soil collected in Pembroke Pines, Florida (25.72231° N, 80.35118° W), using *Microbacterium foliorum* NRRL B-24224. The soil sample (~15 g) was suspended in peptone-yeast extract-calcium (PYCa), incubated at 30°C with shaking for 2 h, and then centrifuged at 2,000 *g*, with the supernatant filtered through a 0.22-µm filter (2). The filtrate was inoculated with *M. foliorum* and enriched for 2 days at 30°C. The enriched sample was filtered and plated in PYCa top agar (0.4%) with *Microbacterium foliorum* as a host. Individual plaques were picked and purified through three rounds of plating. Clear plaques with halo edges measured 4.28 ± 1.36 mm (*N* = 10) in diameter (Fig. 1A). Transmission electron microscopy revealed a podoviral morphology (Fig. 1B), with an icosahedral capsid measuring 65.97 ± 0.47 nm (*N* = 3) in diameter.

DNA was extracted from phage particles in lysate using the Quick-DNA Viral Kit (Zymo). Libraries were prepared using the NEBNext Ultra II DNA Library Prep Kit and sequenced on an Illumina NextSeq 1000 platform (XLEAP P1 kit), generating 1,529,840 single-end, 100-bp reads and approximately 2,600-fold genome coverage. Raw reads were trimmed using cutadapt 4.7 (–nextseq-trim 30), filtered with skewer 0.2.2 (-q 20 -Q 30 -n -l 50) prior to assembly (3, 4), and assembled using Consed v29 (5) and Unicycler v0.5.0 (6), yielding a complete 53,817 bp genome with a guanine–cytosine (GC) content of 60.3% and circularly permuted termini. Genome ends were determined as previously reported (7).

Genome annotation was performed using DNA Master v5.23.6, Glimmer v3.2 (8), GeneMark v3.25 (9), and Starterator v1.2 (https://github.com/SEA-PHAGES/starterator). Putative gene functions were assigned using Phamerator v3.0 (10), BLASTp (11) using the National Center for Biotechnology Information (NCBI) non-redundant and PhagesDB databases, HHpred (12) using PDB_mmCIF70, Pfam-A, UniProt-SwissProt, NCBI Conserved Domains databases, and PECAAN v.20241104 (https://blog.kbrinsgd.org/). Transmembrane domains were predicted using DeepTMHMM (13). ARAGORN v1.2.41 (14) and tRNAscan-SE v2.0 (15) were used to identify tRNA and tmRNA genes, respectively. Default settings were used for all software.

TownLake belongs to class Caudoviricetes and is assigned to subcluster EK1 based on gene content similarity of 35% or higher (16) to genome sequences in the PhagesDB database (17). Its genome contains 52 predicted protein-coding genes including 15 with assigned putative functions, 32 hypothetical proteins, and five predicted membrane proteins. No integrase or immunity repressor genes were identified, suggesting that TownLake is not able to establish lysogeny. No tRNA or tmRNA genes were detected. Genome organization is consistent with other EK phages, with DNA metabolism genes predominantly transcribed on the reverse strand and structural and assembly genes transcribed on the forward strand. TownLake encodes an unusually large and centrally located gene product (gp30) of 4,484 amino acids. Similar large proteins are conserved across EK phages and have been reported to be among the largest gene products identified in actinobacteriophages, yet their biological roles remain unknown (18).

## Data Availability

TownLake is available at GenBank accession no. PV876952 and Sequence Read Archive (SRA) no. SRR33237342.
